# Implementation of a cardiovascular toolkit in primary care increased women Veterans’ engagement in behavior change programs: results from a non-randomized stepped wedge trial

**DOI:** 10.1186/s12875-025-03158-5

**Published:** 2026-01-08

**Authors:** Melissa M. Farmer, Alison B. Hamilton, Erin P. Finley, Martin L. Lee, Catherine Chanfreau, Claire Than, Julian Brunner, C. Amanda Schweizer, Tannaz Moin, Bevanne Bean-Mayberry

**Affiliations:** 1 VA HSR Center for the Study of Healthcare Innovation, Implementation & Policy, Greater Los Angeles Healthcare System, 16111 Plummer Street (152), North Hills, CA 91343 USA; 2 David Geffen School of Medicine at UCLA, Los Angeles, CA USA; 3 Long School of Medicine, University of Texas Health Science Center San Antonio, San Antonio, TX USA; 4 VACO, National Oncology Program, Washington, DC USA

**Keywords:** Health Promotion/Prevention/Screening, Modeling: Multi-level, VA Health Care System, Primary Care, Non-randomized stepped wedge, Veterans, Women

## Abstract

**Background:**

Cardiovascular (CV) disease is the leading cause of death among U.S. women, yet women have a limited understanding of their CV-related morbidity and mortality risks. Provider-, system-, and patient-level barriers point to a need for multi-level evidence-based strategies to facilitate CV risk reduction. Supported by the Replicating Effective Programs implementation strategy, we implemented a CV Toolkit in primary care clinics for women Veterans. The objective was to evaluate the effect of CV Toolkit implementation on participation in behavior change programs that target CV risk reduction.

**Methods:**

In partnership with the Veterans Health Administration (VA) Office of Women’s Health and National Center for Health Promotion and Disease Prevention, we conducted an implementation trial of a CV Toolkit at five geographically diverse VA sites between March 2017-March 2020. Using a non-randomized stepped wedge design, we evaluated the effect of CV Toolkit implementation on participation in the VA MOVE! weight management program, and on participation in health promotion and disease prevention (HPDP) programs (coaching, facilitated groups, etc.) and/or complementary integrative health (CIH) programs (yoga, meditation, etc.). We utilized a three-level (patient, site, time) non-linear fixed effect model with stratification by age (65 and older versus younger). Patient participation, utilization, and demographics were extracted from VA administrative data for all women with at least one primary care visit at a participating site from December 2016-March 2020 (n = 6009).

**Results:**

Women were on average 46 years old; 49% were white, 32% Black, 17% Hispanic; and over a third had CV risk factors and/or mental health diagnoses. For women 65 years and older (n = 540), active toolkit implementation was associated with increased odds of MOVE! participation (OR = 1.09; 95% CI:1.030–1.152) compared to when the toolkit was not active either within or between sites. Women younger than 65 (n = 5469) had increased odds of using HPDP/CIH programs during active toolkit implementation (OR = 1.01; 95% CI:1.002–1.022).

**Conclusions:**

A multilevel intervention and implementation strategy were associated with improved patient-level outcomes—a rarity in implementation trials. Precision implementation may offer important next steps in understanding causality and further specifying how implementation strategies can optimize clinical and implementation outcomes.

**Trial registration:**

Clinical Trials.gov, NCT02991534. Registered 12–09-2016, https://clinicaltrials.gov/study/NCT02991534?cond=NCT02991534&rank=1

## Background

Cardiovascular (CV) disease is the leading cause of death among women in the United States, causing one in three deaths each year when combining heart disease and stroke [1], yet women have a limited understanding of their CV-related morbidity and mortality risks [2]. The American Heart Association (AHA) found a sharp 10-year decline in US women’s knowledge of heart disease as the leading cause of death for American women, from 65% aware in 2009 to 44% in 2019 [2]. In terms of CV risk factors, women suffer disparities in risk factor control (e.g., blood pressure, cholesterol, and intermediate diabetes outcomes) [3–6], and have higher rates of obesity and physical inactivity than men [7], likely contributing to the increasing prevalence of coronary heart disease in women [8–12].

While multiple CV guidelines are available to aid in controlling women’s CV risk factors, addressing CV risk and making health behavior changes are difficult to achieve in routine care due to provider-, system-, and patient-level barriers. Provider- and system-level barriers include lack of time during appointments, lack of awareness of and difficulties interpreting the latest CV prevention guidelines, difficulty accessing relevant electronic medical record data at appointments, lack of electronic tools tracking risk, lack of organized resources for provider communication and referrals, low self-efficacy in counseling about behavior change, habit or inertia, fragmentation of care, complexity of health needs and the perception that patients are not interested or capable of acting on recommendations [6, 13, 14]. Patient-level barriers include limited health literacy, lack of awareness, mixed and confusing messages in the media, beliefs that health is determined by a higher power, difficulties balancing health, finances, physical and mental health conditions, and caretaking responsibilities [13, 15]. These multilevel barriers point to the need for evidence-based strategies to facilitate CV risk reduction.

For Veterans, CV risk prevention and management have been comparable or even better than among the civilian population [16–20], yet sex disparities in CV risk factor control have been identified among Veterans for lipids, blood pressure, and intermediate diabetes outcomes, with women Veterans at elevated risk [21–24]. Between 2000–2015, women Veterans experienced rising rates of hypertension (24 to 27%), hyperlipidemia (15 to 25%), diabetes (8 to 11%), and overweight/obesity (10 to 20%), as well as rising rates of depression (27 to 41%) and post-traumatic stress disorder (PTSD; 6 to 18%) [25, 26], which have been identified as risk factors for CV events among women Veterans [27–30]. As women Veterans are the fastest growing population of Veteran Health Administration (VHA) users [25], the prevalence of both traditional CV risk factors and mental health burden makes addressing CV risk a critical VA priority.

**Fig. 1:**
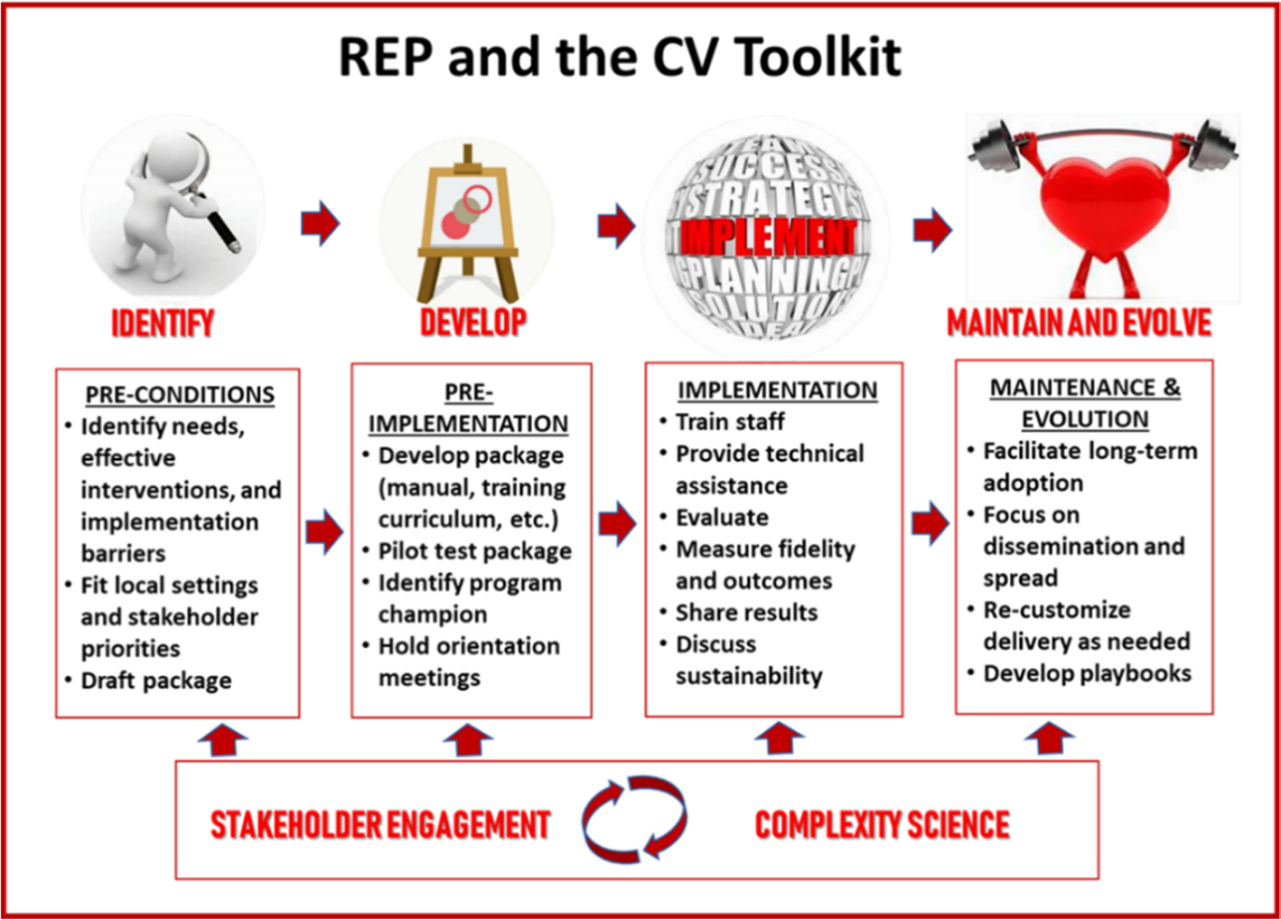
Replicating Effective Programs Implementation Strategy

As part of the Enhancing Mental and Physical Health of Women through Engagement and Retention (EMPOWER) Quality Enhancement Research Initiative (QUERI; QUE 15–272) [31], we conducted an implementation trial of a cardiovascular (CV) toolkit designed to increase engagement in clinical services that focus on CV risk reduction. Implementation was guided by Replicating Effective Programs (REP), a phased evidence-based implementation strategy [32], enhanced with complexity theory [31] and multilevel stakeholder engagement. This combination ensured that our trial embraced the complexity of multilevel systems, worked directly to engage and empower participants across the system, and acted both scientifically and pragmatically to develop tailored-to-context solutions as challenges arose [33]. Formative *pre-conditions work *built off the prior CV Toolkit development, which indicated that evidence-based strategies need to be combined, tailored, and implemented at the local level to facilitate patient activation and provider-patient discussion while providing accountability and support to women to promote CV risk reduction [13]. The *pre-implementation* phase included identifying program priorities in collaboration with our operations partners at the National Center for Health Promotion and Disease Prevention and conducting in-person site visits to engage with local partners (e.g., Primary Care or Women’s Health leaders, frontline providers) and consider local adaptations. This phase included identifying champions and tailoring the toolkit to facilitate incorporation into existing clinic workflow and identify lifestyle/behavioral change programs available at each site, as well as working with sites to determine who would implement each component of the toolkit. During the *implementation phase*, the toolkit was rolled out with REP-guided implementation strategies including training and monthly one-hour technical assistance calls with the research team. During the *maintenance and evolution phase*, we incorporated feedback from sites and disseminated results to site- and national-level partners. See Fig. 1 for illustration of the REP framework and phases of CV Toolkit implementation.

The objective of this paper is to present the results of the implementation trial, which was designed to evaluate the effect of REP-supported CV Toolkit implementation on women Veterans’ participation in behavior change programs targeting CV risk reduction across five VA sites.

## Methods

### Description of the CV toolkit

The CV Toolkit was co-designed with researchers, providers, primary care staff, and VA Women’s Health leadership to increase CV risk identification and documentation, enhance patient-provider communication about CV risk, and increase women Veterans’ participation in relevant evidence-based CV risk reduction services including key VA programs for health behavior changes. The CV Toolkit [13] was an intervention package comprising three key components. The first component was a patient-facing, one-page CV screener, given by the clerk or nurse to women at check-in for their primary care appointment. The CV screener included a series of questions covering personal health history, family history of CV disease, pregnancy/gestational history, smoking status, physical activity, and a final question on what the patient would like to address with her provider at the appointment. The goal was to help make CV risk discussion a priority for women before they enter the exam room. The second component was a provider CV template that mirrored the patient screener to facilitate CV risk discussion and allowed providers to document directly into the electronic health record (EHR). The template provided a summary of data available in the EHR (e.g., last three entries for weight, blood pressure, and cholesterol lab results), open fields allowing providers to enter pertinent information from the discussion, and an action plan section for providers to directly refer patients to relevant programs. Completed templates were accessible to other providers (e.g., to whom the patient has been referred) within the EHR. The third component was a menu of appropriate health promotion/behavioral change programs available at each site, to assist providers with on-the-spot referrals (e.g., MOVE!, Gateway to Healthy Living, etc.).

### Non-randomized stepped wedge study design

The stepped wedge design has been a hallmark of implementation studies for more than 20 years [34]. This design has the advantage, similar to the cross-over design in clinical trials, of allowing for exposure to both a control and experimental intervention, but within each study unit – e.g., site – instead of individual. The control or comparator is the period when the study unit is not being exposed to the experimental intercession. The CV Toolkit evaluation followed a non-randomized stepped wedge design, which relied on a sequential roll-out of the intervention at sites over time [35]. In stepped wedge designs, randomization of the units to the start of the intervention steps is often considered the gold standard in terms of reduced bias and improved efficiency [36]. However, randomization of study units is often not feasible for implementation studies, particularly in “real world” healthcare service settings [37]. To evaluate the impact of the CV Toolkit, we implemented steps within the study based on site readiness. This acknowledges, per complexity theory, that sites are heterogeneous, face multiple constraints, and are not necessarily ready to adopt interventions at times specified by a randomized design.

The non-randomized stepped wedge capitalizes on the use of data collection at all sites over the entire study period both before and after the start of active implementation. All sites begin the observation period in usual care (control state/“off”), and each site moves into active implementation at some point across the study period (implementation state/“on”). In the statistical evaluation, each site serves as a comparator for both itself and for the implementation states of the other sites at each time point: sites that have not yet started active implementation serve as their own control as well as serving as controls for sites already in active implementation. The strength of this design is that the modeling of both comparisons (control to implementation for each site as well as between sites) at each time point helps mitigate concern regarding any site selection bias despite the lack of randomization. Also, by incorporating the actual timing of the implementation into the statistical assessment of the intervention effectiveness, the design accounts for potential threats to validity due to historical trends that may occur outside of the intervention. It also accounts for contextual site characteristics that may affect site implementation and performance.

### Implementation settings

The CV Toolkit was implemented at five VA primary care sites. Sites were eligible if they had at least one women’s health patient-aligned care team (PACT) teamlet (comprised of a primary care provider, registered nurse, licensed vocational or practical nurse, and clerk/scheduler), at least one other clinical staff member (e.g., dietitian, pharmacist or mental health provider, etc.), and administrative staff (Women’s Health Medical Director, Women Veterans Program Manager, and Chief of Primary Care or Primary Care Physician Leader). Sites were selected and recruited through the VA Women’s Health Practice-Based Research Network [38]. The five sites were geographically diverse: two in the West, two in the South, and one in the East. The patient panels at the sites ranged from 470–2205 women.

### Evaluation sample

All women with at least one primary care visit at one of five primary care sites from December 1, 2016 to March 15, 2020 were eligible to receive the CV Toolkit, allowing for a pre-implementation evaluation of six months prior to the first site beginning implementation (June 2017), and implementation concluding with the service pause for COVID-19. All women VA users within these parameters were eligible for the CV Toolkit, regardless of their Veteran status (e.g., non-Veteran women patients who are dependents of disabled Veterans), as all women were potentially at CV risk and could benefit from the CV Toolkit.

### Measures

The primary outcome for the trial was patient participation in VA health behavior change programs that target the reduction of CV risk. To define the outcome, primary care leads and/or lead women’s health providers at each site reviewed a list of common VA program options to confirm availability and identify any additional programs available. This step provided a validated menu of programs available at each site, which was then incorporated into the site’s template. Two specific outcome measures were created, defined as participation in: (1) MOVE!, VA’s long-standing health behavior program focused on weight management, and (2) health promotion and disease prevention (HPDP) and/or complementary integrative health (CIH) approaches [39, 40]. Program access for patients varied by site, with some sites requiring consults by providers and other sites relying on patient self-referrals. Therefore, we created a systematic approach incorporating site-specific consult procedures and codes, patient self-referral options, and VA service line stop codes, and conducted note title searches by key words and individual note review. The primary outcome was measured at the patient level at each site for each month within the evaluation period (1 = participation during the month versus 0 = no participation during the month). Outcome data were measured from December 1, 2016 through August 31, 2020 to include six-month pre-implementation and six-month post-implementation periods for all sites. All data were collected from the VA Corporate Data Warehouse. The project was approved by the VA Central Institutional Review Board.

To identify MOVE! attendance, we used VA service line/stop codes (372 – weight management counseling for individual and 373 – weight management counseling for group), linked consult orders to attendance notes, and searched for additional notes with the key word “move” to capture self-referrals (exclusions for move/movement disorders and bariatric surgery consultations). To capture the first visit of a new MOVE! program, we examined participation trends and found that over 90% of all MOVE! visits took place within 35 days: if a participant continued with the same program, she continued within 35 days. Therefore, we defined that a patient was eligible for a “new” MOVE! program only if the visit was greater than 35 days since a prior MOVE! visit. Participation was coded as any new participation in the month (1 = new MOVE! and 0 = no new MOVE!).

The second outcome measure captured participation in other VA health promotion programs including HPDP (such as Gateway facilitated group, healthy eating, exercise, sleep, personal development, practice positive thinking, etc.) and/or CIH approaches (such as yoga, Tai Chi, mindfulness meditation). We conducted a comprehensive chart review by extracting notes from patients’ EHR. We reviewed these notes and expanded searching key terms for HPDP and CIH attendance in the note titles that were specific to each study site and CV Toolkit template. These key terms included: integrative health, integrative medicine, gateway, tai chi, yoga, fitness, health promotion, mind over body, healing, healthy, and breathing. After the expanded search, one PI and an analyst reviewed the notes and checked the consistency. When there was a discrepancy, the second PI reviewed the note. This step validated that each note was related to delivery of patient care versus something administrative (e.g., no show note). Participation was coded as yes = 1 for participation in at least one program/service (0 = no participation in any program/service).

Patient characteristics including age, race (White, Black, American Indian, Asian, Native Hawaiian, and Unknown/Missing), ethnicity (Hispanic/Latina), and military disability service connection were obtained at the start of the observation period. Medical diagnoses related to CV risk documented in the EHR five years prior to baseline included hypertension, hyperlipidemia, and diabetes; we created an indicator of having at least one of those CV risk factor conditions. We also included a diagnosis of overweight/obesity. For mental health conditions, we included documented depression (e.g., major depressive disorder or depression, unspecified) or PTSD diagnoses in the past five years. Size of site panel of women Veterans was a site-level characteristic.

### Analytic model

Our generalized mixed model included three levels in the hierarchical model: (1) patient, (2) time of intervention (when the site began active implementation of the CV Toolkit overall package), and (3) site. We used hierarchical linear modeling to account for measures at the three different levels of data collection (patient, time of intervention, site) [41], and we utilized the unit-specific model because it provides predictions for the individual rather than the average individual. The three-level mixed model estimates the probability of participation for person *j* at time *i* at site *k,* ${{\boldsymbol{\theta}}}_{{\boldsymbol{i}}{\boldsymbol{j}}{\boldsymbol{k}}}$

1 $$\begin{aligned}{\eta }_{ijk}=&log \left[\frac{{\theta }_{ijk}}{(1-{\theta }_{ijk})}\right]={\gamma }_{000}\\&+{\gamma }_{100}{MONTH\_NUM}_{ijk}\\&+{\gamma }_{200}{TREAT\_CVTool}_{ijk}\\&+{\gamma }_{300}{MONTH\_TREATCVTool}_{ijk}\\&+{\gamma }_{010}{Respondent\_Var1}_{jk}\\&+{\gamma }_{020}{Respondent\_Var2}_{jk}\\&+{\gamma }_{001}{Site\_Var1}_{k}+{\gamma }_{002}{Site\_Var2}_{k}\\&+{r}_{0jk}+{u}_{00k}\end{aligned}$$

2 $$\begin{aligned}{\eta }_{ijk}=&log \left[\frac{{\theta }_{ijk}}{(1-{\theta }_{ijk})}\right]={\gamma }_{000}\\&+{\gamma }_{100}{MONTH\_NUM}_{ijk}\\&+{\gamma }_{200}{TREAT\_CVTool}_{ijk}\\&+{\gamma }_{300}{DURATION}_{ijk}\\&+{\gamma }_{010}{Respondent\_Var1}_{jk}\\&+{\gamma }_{020}{Respondent\_Var2}_{jk}\\&+{\gamma }_{001}{Site\_Var1}_{k}\\&+{\gamma }_{002}{Site\_Var2}_{k}+{r}_{0jk}+{u}_{00k}\end{aligned}$$

One main effect in the model was time (Month 1 -Month 45), which reflects the time trend during which the sites were observed. The second main effect was treatment status (control or active implementation), which indicates if the site was actively implementing or in a control mode (not actively implementing). However, the key independent variable of interest was the time-by treatment interaction, which examines the difference in the time trends for sites in control and active implementation [35]. Results are evaluated in terms of direction of the effect of the CV Toolkit being “on” (active) as well as the significance, i.e., a significant positive interaction effect indicated an increase over time in the outcome (participation), comparing sites in active implementation versus not actively implementing. In the model, respondent and observation are random effects and contribute to the error term, ${r}_{0jk}+{u}_{00k}$, respectively. The other independent variables (site, time, duration and treatment status) are fixed effects. We used robust standard errors to allow for fewer restrictions on the cluster variability assumption. Based on the skewed distribution in the participation outcomes by age, all models were estimated stratified by age: women younger than 65 years old and women aged 65 and older. Models were estimated using HLM software (HLM7, 2024) [42].

The power analysis was based on a 3-level hierarchical linear model where patients (level 1) are clustered within time steps (level 2), clustered within sites (level 3). There were nine items considered: α: 0.05; target power (1- β): 0.80; 60%; probability of outcome for control: 50%; plausibility for retention for patients who do not receive the intervention: 5%−85%; Effect Size Variability: 1%; number of sites: 4; number of time steps per site: 17; and number of patients per time step: 65. These considerations resulted in a sample size of 6009, but the degrees of freedom for our analysis was based on the number of sites. Note that we exceeded the number of sites (5 instead of required 4) and the number of time steps (44 instead of 17), which makes this calculation conservative.

## Results

**Fig. 2:**
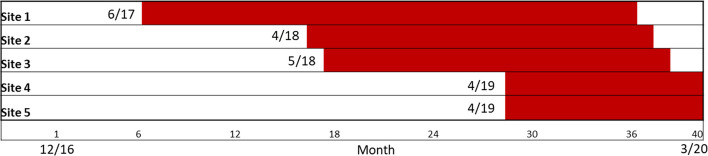
Non-Randomized Stepped Wedge Implementation Schedule

Following the non-randomized stepped wedge design, the first site launched in June 2017, and the final site ended in March 2020 (see Fig. 2). All sites achieved CV Toolkit uptake during active implementation; findings of a mixed-method evaluation assessing uptake have been previously published [43]. Implementation duration varied across sites from 11–29 months. Two sites ended early in March 2020 due to the cessation of in-person primary care services because of the COVID-19 pandemic.

A total of 6009 women had at least one primary care visit over the study period. The average age of women was 46 years, and 49% of women were white, 32% Black, and 17% Hispanic/Latina. The younger cohort (less than 65 years of age) had higher percentages of Black and Hispanic women compared to the older cohort. Eleven percent of women had diabetes, 30% high cholesterol, 26% hypertension, 41% had at least one of those CV risk factors, and the older cohort had significantly higher rates for all conditions. Twenty-nine percent of women were diagnosed as overweight/obesity with no significant differences across age cohorts. For mental health, 42% had a depression diagnosis, 33% had a PTSD diagnosis, and both mental health diagnoses were more predominant among the younger women. Seventy-five percent of the women had a service-connected disability (78% of the younger cohort compared to 49% of the older cohort). Most women (96%) seen at the sites were Veterans (Table 1).

**Table 1:** Characteristics of women seen in primary care at the five implementation sites

		Women younger	Women 65	
	Total Sample	than age 65	years & older	
Characteristic	(N = 6009)	(N = 5469)	(N = 540)	P-value
Mean age (SD)	45.6 (14.7)	43.0 (12.4)	72.6 (7.5)	< 0.0001
Race				< 0.0001
White	2954 (49.2%)	2630 (48.1%)	324 (60.0%)	
Black	1905 (31.7%)	1768 (32.3%)	137 (25.4%)	
Asian, NH, OPI, AI or, AN*	376 (6.3%)	358 (6.6%)	18 (3.3%)	
Unknown	774 (12.9%)	713 (13.0%)	61 (11.3%)	
Ethnicity				< 0.0001
Hispanic or Latina	1011 (16.8%)	973(17.8%)	38 (7.0%)	
Not Hispanic or Latina	4742 (78.9%)	4265 (78.0%)	477 (88.3%)	
Unknown	256 (4.3%)	231 (4.2%)	25 (4.6%)	
Diagnosis in last 5 years				
Diabetes	677 (11.3%)	521 (9.5%)	156 (28.9%)	< 0.0001
High cholesterol	1786 (29.7%)	1415 (25.9%)	371 (68.7%)	< 0.0001
Hypertension	1585 (26.4%)	1207 (22.1%)	378 (70.0%)	< 0.0001
Has at least 1 of 3 CV risk factors**	2432 (40.5%)	1968 (36.0%)	464 (85.9%)	< 0.0001
Overweight/Obesity	1769 (29.4%)	1595 (29.2%)	174 (32.2%)	0.14
Mental Health diagnosis in last 5 years				
Depression	2503 (41.7%)	2318 (42.4%)	185 (34.3%)	< 0.0001
PTSD	1953 (32.5%)	1853 (33.9%)	100 (18.5%)	< 0.0001
Service Connected	4515 (75.1%)	4250 (77.7%)	265 (49.1%)	< 0.0001
Veteran	5757 (95.8%)	5221 (95.5%)	536 (99.3%)	< 0.0001

^*^Asian, Native Hawaiian, Other Pacific Islander, American Indian, or Alaska Native

^**^At least one of the following CV risk factors: hypertension, high cholesterol, and diabetes

Results from the non-randomized stepped wedge model for each outcome stratified by age are shown in Table 2. For MOVE! participation for women 65 years and older, the interaction effect was significant, indicating that in sites with the CV Toolkit active, women 65 years and older had greater odds (OR = 1.09; CI:1.030,1.152) of participating in the MOVE! program than in sites without the CV Toolkit active (Table 2). For women younger than age 65, the effect was not significant (OR = 1.00; CI:0.976,1.022). In contrast, women less than 65 years old who were seen at sites with the CV Toolkit active had greater odds of participation (OR = 1.01; CI = 1.002–1.022) in HPDP and/or CIH services, compared to women in inactive sites [35]. We conducted a series of sensitivity analyses by testing site-level indicators correlated with variations in uptake (e.g., site size, number of providers using CV Toolkit, and implementation duration), and the overall results did not change.

**Table 2:** Intervention effectiveness stratified by age^a^

**Attendance at MOVE! Exercise Program for women age 65 and older**			
	Odds Ratio	Confidence Interval	*P* value
Intercept	0.01	(0.005,0.009)	< 0.001
Time	0.87	(0.838,0.908)	< 0.001
CV Toolkit Intervention	1.74	(0.707,4.280)	0.228
Interaction	1.09	(1.030,1.152)	0.003

**Attendance at MOVE! Exercise Program for women less than 65 years old**			
	Odds Ratio	Confidence Interval	*P* value
Intercept	0.00	(0.002,0.007)	< 0.001
Time	0.99	(0.974,1.003)	0.114
CV Toolkit Intervention	1.28	(0.820,1.985)	0.28
Interaction	1.00	(0.976,1.022)	0.91

**Attendance at HPDP Programs and/or CIH service use for women age 65 and older**			
	Odds Ratio	Confidence Interval	*P* value
Intercept	0.00	(0.001,0.016)	< 0.001
Time	0.96	(0.892,1.040)	0.34
CV Toolkit Intervention	0.39	(0.066,2.297)	0.298
Interaction	1.06	(0.959,1.165)	0.265

**Attendance at HPDP Programs and/or CIH service use for women less than 65 years old**			
	Odds Ratio	Confidence Interval	*P* value
Intercept	0.01	(0.003,0.027)	< 0.001
Time	1.00	(0.993,1.010)	0.715
CV Toolkit Intervention	0.79	(0.653,0.953)	0.014
Interaction	1.01	(1.002,1.022)	0.015

^a^Fixed effects (Unit-specific model) with robust standard errors

## Discussion

The CV Toolkit intervention was implemented in five diverse clinical sites. Active implementation of the CV Toolkit was significantly associated with increased participation in behavior change programs. While improvements in participation were found for both age groups, significant improvements varied by program type. Specifically, women Veterans who were age 65 and older had greater odds of participating in the MOVE! program in sites when the CV Toolkit was active compared to women at sites when the CV Toolkit was not active, while younger women had greater odds of participating in HPDP/CIH approaches that often include physical activity and stress reduction. Results suggest that program variety may be key to supporting CV targeted behavior change for women across the life span. Furthermore, our program successfully illustrates the importance of ensuring, per the American Heart Association, that patients receive primary care-based services that address CV risk [44].

Our findings of differential program participation by age also underscore the shifting needs and preferences of women Veterans across the life course [46], and may help to illustrate how VA and comparable large-scale healthcare systems can benefit from a learning health systems approach [47]. Implementation and cohort studies with women Veterans over the past decade have demonstrated that VA can adapt, expand, and respond to new challenges, including growing cohorts of aging Veterans [48–50]. This knowledge is important for ensuring a more responsive health system [51].

Our study stands out for its positive, patient-level impacts on participation in behavior change programs [45]. Despite significant variation in clinics’ geographic location and panel size, implementation with enhanced REP achieved adoption of the CV Toolkit at all five participating sites. Our study demonstrates that REP-guided implementation can help facilitate program uptake, service delivery improvements, and patient-level engagement in real-world settings.

### Limitations and strengths

Our findings should be interpreted with several limitations. First, the implementation sites care for a diverse and varied number of women Veterans; however, the analytic sample for the model was limited to five sites. The non-randomized stepped wedge accommodates for time trend and history, but implementation start timing for each site was not randomized, which may have introduced bias not accounted for in the model. Also, the number of sites (n = 5) restricted the degrees of freedom in the statistical model, which meant we were limited in the number of covariates we could examine in the final models. An additional limitation was the early termination of implementation at two sites due to COVID-19. One site limited care to urgent needs and one site closed during the pandemic, resulting in an early close to the implementation trial. This meant that two sites had reduced implementation periods and likely had lower uptake than if they had continued the full time. During the COVID-19 period, primary care and health promotion programs were modified in all sites, and health promotion and behavior change programs were often delayed during the transition to virtual options. Therefore, post-implementation data reflect these unanticipated and unprecedented changes. Another limitation is that for programs requiring a consult order, scheduling by phone could have led to errors or losses to follow-up scheduling, which would likely bias our results to the null. Finally, we did not measure variation in uptake of individual CV Toolkit components, nor was the study designed to examine the effectiveness of individual components. Specifically, the patient-facing screener was administered by the site and not collected for research and provider-patient discussions that did not result in a completed template were not recorded. Despite these challenges, we found significant associations between active implementation and health behavior participation within age cohorts.

A strength of this work is it represents a learning health system in action [52]. First, national operations partners (VA Office of Women’s Health) identified a clear need: to address high CV risk among women Veterans. Then, collaboration between researchers and operations partners led to the identification of organizational-, provider- and patient-level barriers to and facilitators of CV risk identification, documentation, discussion, and reduction, which in turn led to the development of the CV Toolkit as a package of focused clinical tools [13]. The VA Quality Enhancement Research Initiative [53] then funded the implementation of the CV Toolkit into practice, where we achieved the outcome of improved engagement in focused health behavior change programs.

## Conclusions

Refining, implementing, and evaluating the innovative CV Toolkit to facilitate appropriate screening, discussion, and documentation of CV risk led to actionable steps to manage risk factors. CV Toolkit use at the clinic level helped improve engagement and retention of women Veterans in patient-centered, proactive, personalized care to address CV risk. In prior EMPOWER analyses, we have found that women prefer to engage in interventions that are convenient and sensitive to their life circumstances, especially since a substantial proportion of women are isolated from care due to competing responsibilities, rurality and/or urban isolation, as well as disabilities, histories of trauma, or sex bias [13, 50, 54–56]. Given that we saw significant differences in health behavior change program engagement by age cohort, future research is needed to understand whether other patient characteristics influence program choice and whether and how these choices or preferences change through the life course.

Our implementation trial directly examined patient-level participation in clinical services, which is rare in implementation studies [45]. We found that a multilevel intervention and enhanced REP implementation strategy were associated with improved patient-level outcomes when embedded in primary care. Precision implementation [57] may offer important next steps in understanding causality and further specifying how implementation strategies can optimize both clinical and implementation outcomes.

## Data Availability

The datasets generated and analyzed during the current study are not publicly available because data was obtained from the Veterans Health Administration electronic health records. Sharing data would violate individual privacy.

## Abbreviations

CV: Cardiovascular
CIH: Complementary integrative health
EHR: Electronic health record
EMPOWER: Enhancing Mental and Physical Health of Women through Engagement and Retention
HPDP: Health promotion and disease prevention
HLM: Hierarchical linear Modeling
PACT: Patient-aligned care team
QUERI: Quality Enhancement Research Initiative
REP: Replicating Effective Programs
VA: Veteran Health Administration

## References

[1] About Women and Heart Disease | Heart Disease | CDC. Accessed September 24, 2025. https://www.cdc.gov/heart-disease/about/women-and-heart-disease.html

[2] Cushman M, Shay CM, Howard VJ, et al. Ten-Year Differences in Women’s Awareness Related to Coronary Heart Disease: Results of the 2019 American Heart Association National Survey: A Special Report From the American Heart Association. Circulation. Published online September 21, 2020:CIR0000000000000907. 10.1161/CIR.0000000000000907 10.1161/CIR.0000000000000907 PMC11181805 32954796

[3] Arora S, Stouffer GA, Kucharska-Newton AM, et al. Twenty year trends and sex differences in young adults hospitalized with acute myocardial infarction. Circulation. 2019;139(8):1047–56. 10.1161/CIRCULATIONAHA.118.037137. 30586725 10.1161/CIRCULATIONAHA.118.037137 PMC6380926

[4] Muiesan ML, Salvetti M, Rosei CA, Paini A. Gender Differences in Antihypertensive Treatment: Myths or Legends? High Blood Press Cardiovasc Prev Off J Ital Soc Hypertens. 2016;23(2):105–13. 10.1007/s40292-016-0148-1. 10.1007/s40292-016-0148-1 27106810

[5] Bird CE, Elliott MN, Adams JL, et al. How do gender differences in quality of care vary across Medicare Advantage plans? J Gen Intern Med. 2018;33(10):1752–9. 10.1007/s11606-018-4605-5. 30097976 10.1007/s11606-018-4605-5 PMC6153209

[6] Vogel B, Acevedo M, Appelman Y, et al. The Lancet women and cardiovascular disease commission: reducing the global burden by 2030. Lancet. 2021;397(10292):2385–438. 10.1016/S0140-6736(21)00684-X. 34010613 10.1016/S0140-6736(21)00684-X

[7] Mayo X, Liguori G, Iglesias-Soler E, et al. The active living gender’s gap challenge: 2013–2017 Eurobarometers physical inactivity data show constant higher prevalence in women with no progress towards global reduction goals. BMC Public Health. 2019;19(1):1677. 10.1186/s12889-019-8039-8. 31830956 10.1186/s12889-019-8039-8 PMC6909566

[8] Benjamin EJ, Muntner P, Alonso A, et al. Heart disease and stroke statistics-2019 update: a report from the American Heart Association. Circulation. 2019;139(10):e56–528. 10.1161/CIR.0000000000000659. 30700139 10.1161/CIR.0000000000000659

[9] Breland JY, Phibbs CS, Hoggatt KJ, et al. The obesity epidemic in the Veterans Health Administration: prevalence among key populations of women and men veterans. J Gen Intern Med. 2017;32(Suppl 1):11–7. 10.1007/s11606-016-3962-1. 28271422 10.1007/s11606-016-3962-1 PMC5359156

[10] Engberding N, Wenger NK. Management of hypertension in women. Hypertens Res Off J Jpn Soc Hypertens. 2012;35(3):251–60. 10.1038/hr.2011.210. 10.1038/hr.2011.210 22158115

[11] Haskell SG, Brandt C, Burg M, et al. Incident Cardiovascular Risk Factors Among Men and Women Veterans After Return From Deployment. Med Care. 2017;55(11):948–55. 10.1097/MLR.0000000000000801. 28984707 10.1097/MLR.0000000000000801

[12] Virani SS, Alonso A, Benjamin EJ, et al. Heart disease and stroke statistics-2020 update: a report from the American Heart Association. Circulation. 2020;141(9):e139–596. 10.1161/CIR.0000000000000757. 31992061 10.1161/CIR.0000000000000757

[13] Bean-Mayberry B, Moreau J, Hamilton AB, et al. Cardiovascular risk screening among women veterans: identifying provider and patient barriers and facilitators to develop a clinical toolkit. Womens Health Issues. 2022;32(3):284–92. 10.1016/j.whi.2021.12.003. 35115227 10.1016/j.whi.2021.12.003

[14] Taylor K, Kane M, Bail J. Barriers to screening, diagnosis, and prevention of cardiovascular disease in women veterans. Mil Med. 2025. 10.1093/milmed/usaf229. 40434423 10.1093/milmed/usaf229

[15] Magnani JW, Mujahid MS, Aronow HD, et al. Health Literacy and Cardiovascular Disease: Fundamental Relevance to Primary and Secondary Prevention: A Scientific Statement From the American Heart Association. Circulation. Published online July 2018. 10.1161/CIR.0000000000000579 10.1161/CIR.0000000000000579 PMC6380187 29866648

[16] O’Hanlon C, Huang C, Sloss E, et al. Comparing VA and Non-VA Quality of Care: A Systematic Review. J Gen Intern Med. 2017;32(1):105–21. 10.1007/s11606-016-3775-2. 27422615 10.1007/s11606-016-3775-2 PMC5215146

[17] Anhang Price R, Sloss EM, Cefalu M, Farmer CM, Hussey PS. Comparing quality of care in Veterans Affairs and non-Veterans Affairs settings. J Gen Intern Med. 2018;33(10):1631–8. 10.1007/s11606-018-4433-7. 29696561 10.1007/s11606-018-4433-7 PMC6153237

[18] Controlling High Blood Pressure - Quality of Care. Accessed September 27, 2020. https://www.va.gov/QUALITYOFCARE/initiatives/compare/high-blood-pressure-control.asp

[19] Cardiovascular Care – Controlling High Cholesterol - Quality of Care. Accessed September 27, 2020. https://www.va.gov/QUALITYOFCARE/initiatives/compare/cardiovascular-care-controlling-cholesterol-levels.asp

[20] Mei P, Cotiga D, Mahana I, et al. Cardiovascular care in women veterans: an updated profile. Curr Cardiol Rep. 2025;27(1):93. 10.1007/s11886-025-02247-2. 40504328 10.1007/s11886-025-02247-2

[21] Lardizabal JA, Deedwania PC. Benefits of statin therapy and compliance in high risk cardiovascular patients. Vasc Health Risk Manag. 2010;6:843–53. 10.2147/VHRM.S9474. 20957130 10.2147/VHRM.S9474 PMC2952453

[22] Chou AF, Wong L, Weisman CS, et al. Gender disparities in cardiovascular disease care among commercial and medicare managed care plans. Womens Health Issues Off Publ Jacobs Inst Womens Health. 2007;17(3):139–49. 10.1016/j.whi.2007.03.004. 10.1016/j.whi.2007.03.004 17481918

[23] Farmer MM, Rose DE, Riopelle D, Lanto AB, Yano EM. Gender differences in smoking and smoking cessation treatment: an examination of the organizational features related to care. Womens Health Issues Off Publ Jacobs Inst Womens Health. 2011;21(4 Suppl):S182-189. 10.1016/j.whi.2011.04.018. 10.1016/j.whi.2011.04.018 21724139

[24] Nanna MG, Wang TY, Xiang Q, et al. Sex differences in the use of statins in community practice. Circ Cardiovasc Qual Outcomes. 2019;12(8):e005562. 10.1161/CIRCOUTCOMES.118.005562. 31416347 10.1161/CIRCOUTCOMES.118.005562 PMC6903404

[25] Frayne S, Phibbs C, Saechao F, et al. Sourcebook: women veterans in the veterans health administration, volume 4, longitudinal trends in sociodemographics, utilization, health profile, and geographic distribution. Washington DC: Women’s Health Services, Veterans Health Administraton, Department of Veterans Affairs; 2018. p. 144.

[26] Whitehead AM, Maher NH, Goldstein K, et al. Sex differences in veterans’ cardiovascular health. J Womens Health. 2019;28(10):1418–27. 10.1089/jwh.2018.7228. 10.1089/jwh.2018.7228 30839237

[27] Creech SK, Pulverman CS, Crawford JN, et al. Clinical Complexity in Women Veterans: A Systematic Review of the Recent Evidence on Mental Health and Physical Health Comorbidities. Behav Med Wash DC. Published online August 12, 2019:1–19. 10.1080/08964289.2019.1644283 10.1080/08964289.2019.1644283 31403895

[28] Ebrahimi R, Lynch KE, Beckham JC, et al. Association of posttraumatic stress disorder and incident ischemic heart disease in women veterans. JAMA Cardiol. 2021;6(6):642–51. 10.1001/jamacardio.2021.0227. 33729463 10.1001/jamacardio.2021.0227 PMC7970390

[29] Han JK, Yano EM, Watson KE, Ebrahimi R. Cardiovascular care in women veterans. Circulation. 2019;139(8):1102–9. 10.1161/CIRCULATIONAHA.118.037748. 30779640 10.1161/CIRCULATIONAHA.118.037748

[30] Jeon-Slaughter H, Chen X, Tsai S, Ramanan B, Ebrahimi R. Developing an internally validated Veterans Affairs women cardiovascular disease risk score using Veterans Affairs national electronic health records. J Am Heart Assoc. 2021;10(5):e019217. 10.1161/JAHA.120.019217. 33619994 10.1161/JAHA.120.019217 PMC8174271

[31] Hamilton AB, Farmer MM, Moin T, et al. Enhancing Mental and Physical Health of Women through Engagement and Retention (EMPOWER): a protocol for a program of research. Implement Sci IS. 2017;12(1):127. 10.1186/s13012-017-0658-9. 29116022 10.1186/s13012-017-0658-9 PMC5678767

[32] Powell BJ, Fernandez ME, Williams NJ, et al. Enhancing the impact of implementation strategies in healthcare: a research agenda. Front Public Health. 2019. 10.3389/fpubh.2019.00003. 30723713 10.3389/fpubh.2019.00003 PMC6350272

[33] Reed JE, Howe C, Doyle C, Bell D. Successful healthcare improvements from translating evidence in complex systems (SHIFT-Evidence): simple rules to guide practice and research. Int J Qual Health Care. 2019;31(3):238–44. 10.1093/intqhc/mzy160. 30085160 10.1093/intqhc/mzy160 PMC6464098

[34] Hussey MA, Hughes JP. Design and analysis of stepped wedge cluster randomized trials. Contemp Clin Trials. 2007;28(2):182–91. 10.1016/j.cct.2006.05.007. 16829207 10.1016/j.cct.2006.05.007

[35] Huynh AK, Lee ML, Farmer MM, Rubenstein LV. Application of a nonrandomized stepped wedge design to evaluate an evidence-based quality improvement intervention: a proof of concept using simulated data on patient-centered medical homes. BMC Med Res Methodol. 2016;16(1):143. 10.1186/s12874-016-0244-x. 27769177 10.1186/s12874-016-0244-x PMC5073914

[36] Hu Y, Hoover DR. Non-randomized and randomized stepped-wedge designs using an orthogonalized least squares framework. Stat Methods Med Res. 2018;27(4):1202–18. 10.1177/0962280216657852. 27405326 10.1177/0962280216657852 PMC5225259

[37] West SG, Duan N, Pequegnat W, et al. Alternatives to the randomized controlled trial. Am J Public Health. 2008;98(8):1359–66. 10.2105/AJPH.2007.124446. 18556609 10.2105/AJPH.2007.124446 PMC2446460

[38] Pomernacki A, Carney DV, Kimerling R, et al. Lessons from Initiating the First Veterans Health Administration (VA) Women’s Health Practice-based Research Network (WH-PBRN) Study. J Am Board Fam Med JABFM. 2015;28(5):649–57. 10.3122/jabfm.2015.05.150029. 26355137 10.3122/jabfm.2015.05.150029

[39] Braun K, Erickson M, Utech A, List R, Garcia JM. Evaluation of veterans MOVE! program for weight loss. J Nutr Educ Behav. 2016;48(5):299-303.e1. 10.1016/j.jneb.2016.02.012. 27169639 10.1016/j.jneb.2016.02.012

[40] Farmer MM, McGowan M, Yuan AH, Whitehead AM, Osawe U, Taylor SL. Complementary and Integrative Health Approaches Offered in the Veterans Health Administration: Results of a National Organizational Survey. J Altern Complement Med N Y N. 2021;27(S1):S124–30. 10.1089/acm.2020.0395. 10.1089/acm.2020.0395 33788607

[41] Woltman H, Feldstain A, MacKay JC, Rocchi M. An introduction to hierarchical linear modeling. Tutor Quant Methods Psychol. 2012;8(1):52–69. 10.20982/tqmp.08.1.p052.

[42] HLM – Scientific Software International, Inc. Accessed September 22, 2024. https://ssicentral.com/index.php/products/hlm-general/

[43] Brunner J, Farmer MM, Bean-Mayberry B, et al. Implementing clinical decision support for reducing women Veterans’ cardiovascular risk in VA: a mixed-method, longitudinal study of context, adaptation, and uptake. Front Health Serv. 2022;2:946802. 10.3389/frhs.2022.946802. 36925876 10.3389/frhs.2022.946802 PMC10012802

[44] Laddu D, Ma J, Kaar J, et al. Health behavior change programs in primary care and community practices for cardiovascular disease prevention and risk factor management among midlife and older adults: a scientific statement from the American Heart Association. Circulation. 2021;144(24):e533–49. 10.1161/CIR.0000000000001026. 34732063 10.1161/CIR.0000000000001026 PMC9188324

[45] Proctor EK, Bunger AC, Lengnick-Hall R, et al. Ten years of implementation outcomes research: a scoping review. Implement Sci. 2023;18(1):31. 10.1186/s13012-023-01286-z. 37491242 10.1186/s13012-023-01286-z PMC10367273

[46] Sheahan KL, Goldstein KM, Than CT, et al. Women veterans’ healthcare needs, utilization, and preferences in veterans affairs primary care settings. J Gen Intern Med. 2022;37(Suppl 3):791–8. 10.1007/s11606-022-07585-3. 36042076 10.1007/s11606-022-07585-3 PMC9481772

[47] Kilbourne AM, Jones PL, Atkins D. Accelerating implementation of research in learning health systems: lessons learned from VA health services research and NCATS clinical science translation award programs. J Clin Transl Sci. 2020;4(3):195–200. 10.1017/cts.2020.25. 32695488 10.1017/cts.2020.25 PMC7348004

[48] Marshall V, Stryczek KC, Haverhals L, et al. The focus they deserve: improving women veterans’ health care access. Womens Health Issues. 2021;31(4):399–407. 10.1016/j.whi.2020.12.011.33582001 10.1016/j.whi.2020.12.011

[49] Washington DL, Bean-Mayberry B, Riopelle D, Yano EM. Access to care for women veterans: delayed healthcare and unmet need. J Gen Intern Med. 2011;26 Suppl 2(Suppl 2):655–661. 10.1007/s11606-011-1772-z 10.1007/s11606-011-1772-z PMC3191223 21989618

[50] Dyer KE, Moreau JLP, Finley EP, et al. Tailoring an evidence-based lifestyle intervention to meet the needs of women Veterans with prediabetes. Women Health. 2020;60(7):748–62. 10.1080/03630242.2019.1710892. 31959089 10.1080/03630242.2019.1710892 PMC8435559

[51] Golden RE, Klap R, Carney DV, et al. Promoting learning health system feedback loops: Experience with a VA practice-based research network card study. Healthc Amst Neth. 2021;8 Suppl 1(Suppl 1):100484. 10.1016/j.hjdsi.2020.100484 10.1016/j.hjdsi.2020.100484 PMC8920519 34175097

[52] Guise JM, Savitz LA, Friedman CP. Mind the gap: putting evidence into practice in the era of learning health systems. J Gen Intern Med. 2018;33(12):2237–9. 10.1007/s11606-018-4633-1. 30155611 10.1007/s11606-018-4633-1 PMC6258636

[53] Kilbourne AM, Goodrich DE, Miake-Lye I, Braganza MZ, Bowersox NW. Quality enhancement research initiative implementation roadmap: toward sustainability of evidence-based practices in a learning health system. Med Care. 2019;57:S286–93. 10.1097/MLR.0000000000001144. 31517801 10.1097/MLR.0000000000001144 PMC6750196

[54] Mattocks K, Casares J, Brown A, et al. Women veterans’ experiences with perceived gender bias in U.S. department of veterans affairs specialty care. Womens Health Issues. 2020;30(2):113–9. 10.1016/j.whi.2019.10.003. 31735581 10.1016/j.whi.2019.10.003

[55] McBain SA, Garneau-Fournier J, Turchik JA. The relationship between provider gender preferences and perceptions of providers among veterans who experienced military sexual trauma. J Interpers Violence. 2022;37(5–6):NP2868–90. 10.1177/0886260520944536. 32741237 10.1177/0886260520944536

[56] Klap R, Darling JE, Hamilton AB, et al. Prevalence of stranger harassment of women veterans at Veterans Affairs medical centers and impacts on delayed and missed care. Womens Health Issues. 2019;29(2):107–15. 10.1016/j.whi.2018.12.002. 30686577 10.1016/j.whi.2018.12.002

[57] Frank HE, Kemp J, Benito KG, Freeman JB. Precision implementation: an approach to mechanism testing in implementation research. Adm Policy Ment Health. 2022;49(6):1084–94. 10.1007/s10488-022-01218-x. 36167942 10.1007/s10488-022-01218-x

